# Natural polymorphisms in HIV-1 CRF01_AE strain and profile of acquired drug resistance mutations in a long-term combination treatment cohort in northeastern China

**DOI:** 10.1186/s12879-020-4808-3

**Published:** 2020-02-26

**Authors:** Zesong Sun, Jinming Ouyang, Bin Zhao, Minghui An, Lin Wang, Haibo Ding, Xiaoxu Han

**Affiliations:** 1 NHC Key Laboratory of AIDS Immunology (China Medical University), Department of Laboratory Medicine, The First Affiliated Hospital of China Medical University, Shenyang, 110001 China; 2 National Clinical Research Center for Laboratory Medicine, The First Affiliated Hospital of China Medical University, Shenyang, 110001 China; 3 Key Laboratory of AIDS Immunology, Chinese Academy of Medical Sciences, Shenyang, 110001 China; 4 Collaborative Innovation Center for Diagnosis and Treatment of Infectious Diseases, 79 Qingchun Street, Hangzhou, 310003 China

**Keywords:** HIV-1, CRF01_AE, Polymorphism, Drug resistance mutation, Co-variation, Deep sequencing

## Abstract

**Background:**

The impacts of genetic polymorphisms on drug resistance mutations (DRMs) among various HIV-1 subtypes have long been debated. In this study, we aimed to analyze the natural polymorphisms and acquired DRM profile in HIV-1 CRF01_AE-infected patients in a large first-line antiretroviral therapy (ART) cohort in northeastern China.

**Methods:**

The natural polymorphisms of CRF01_AE were analyzed in 2034 patients from a long-term ART cohort in northeastern China. The polymorphisms in 105 treatment failure (TF) patients were compared with those in 1148 treatment success (TS) patients. The acquired DRM profile of 42 patients who experienced TF with tenofovir/lamivudine/efavirenz (TDF/3TC/EFV) treatment was analyzed by comparing the mutations at TF time point to those at baseline. The Stanford HIVdb algorithm was used to interpret the DRMs. Binomial distribution, McNemar test, Wilcoxon test and CorMut package were used to analyze the mutation rates and co-variation. Deep sequencing was used to analyze the evolutionary dynamics of co-variation.

**Results:**

Before ART, there were significantly more natural polymorphisms of 31 sites on reverse transcriptase (RT) in CRF01_AE than subtype B HIV-1 (|Z value| ≥ 3), including five known drug resistance-associated sites (238, 118, 179, 103, and 40). However, only the polymorphism at site 75 was associated with TF (|Z value| ≥ 3). The mutation rate at 14 sites increased significantly at TF time point compared to baseline, with the most common DRMs comprising G190S/C, K65R, K101E/N/Q, M184 V/I, and V179D/I/A/T/E, ranging from 66.7 to 45.2%. Moreover, two unknown mutations (V75 L and L228R) increased by 19.0 and 11.9% respectively, and they were under positive selection (Ka/Ks > 1, log odds ratio [LOD] > 2) and were associated with several other DRMs (cKa/Ks > 1, LOD > 2). Deep sequencing of longitudinal plasma samples showed that L228R occurred simultaneously or followed the appearance of Y181C.

**Conclusion:**

The high levels of natural polymorphisms in CRF01_AE had little impact on treatment outcomes. The findings regarding potential new CRF01_AE-specific minor DRMs indicate the need for more studies on the drug resistance phenotype of CRF01_AE.

## Background

At the end of 2017, there were approximately 36.9 million people living with HIV, 59% of whom were receiving antiretroviral therapy (ART) [1], which significantly reduces morbidity and mortality but requires lifelong treatment. Moreover, some people experience treatment failure (TF) because of drug resistance [2]. A multicenter retrospective cohort study of 1926 patients who failed first-line regimens from 36 countries between 1998 to 2015 showed that the drug resistance rate at TF was 20–35% in Europe and North America, 39% in Asia and up to 57% in sub-Saharan Africa [3]. Drug-resistant strains can also spread to treatment-naïve patients, causing HIV transmitted drug resistance [4]. A study of 4140 treatment-naïve newly diagnosed HIV-infected individuals from 26 European countries between 2008 and 2010 showed that the overall prevalence of transmitted drug resistance was 8.3% and it did not change significantly over time [5]. However, it is increasing at a substantial rate in low- and middle-income countries (LMICs). This is especially true regarding the drug resistance rate to non-nucleoside reverse transcriptase inhibitors (NNRTIs), which was about 4% in Asia and near 10% in southern and eastern Africa and Latin America in 2016, the threshold used by the World Health Organization (WHO) to determine when to change first-line ART regimens [6].

Most drug resistance genotype data are derived from subtype B HIV-1, which is responsible for about 12% of infections worldwide and is mainly epidemic in North America and Europe [7]. However, non-B HIV-1 strains demonstrate many genetic differences from subtype B, and this might enable different types and rates of drug resistance mutations (DRMs) to the same drugs [8–10]. Both in vitro and in vivo studies have shown inconsistencies in DRMs between subtype B and some non-B HIV-1 strains [11, 12]. For example, V90I and V179E occurred more frequently after treatment with etravirine (ETR) and rilpivirine (RPV) in non-B HIV-1 compared to subtype B HIV-1 [13]. It has been suggested that the Stanford HIVdb algorithm derived from subtype B HIV-1 might not be completely applicable to non-B HIV-1, and more studies are needed on the genetic polymorphisms and DRM characteristics of non-B HIV-1 strains.

CRF01_AE is the first reported circulating recombinant form (CRF) of HIV-1 and one of the most influential CRFs in the world [14], accounting for 5.3% of the total HIV-1 infections worldwide and increasing over time [7]. CRF01_AE accounts for about 80% of CRFs in southeast and east Asia, and it increased consistently in east Asia between 2010 to 2015 [7]. In China, CRF01_AE is one of the main epidemic strains of HIV-1, accounting for 42.5% of the reported HIV-1 infection cases in China according to a systematic review [15], and multiple lineages of CRF01_AE have been reported to be transmitted in China [16, 17].

The DRM characteristics of CRF01_AE have been reported in several cross-sectional studies. However, most of these population-based studies assessed the prevalence of transmitted or acquired DRMs among populations [18–21] or compared the mutation rates between different populations [22, 23]. Few studies have evaluated the associations between polymorphisms and treatment outcomes. Even fewer studies have performed self-control analyses in ART cohorts and evaluations of the correlations between various mutations.

In this study, we analyzed the natural polymorphisms of CRF01_AE from a large ART cohort in northeastern China and compared the polymorphisms between patients who experienced TF and those who experienced treatment success (TS). The acquired DRM profile was determined using self-control analyses that involved comparing baseline data to data collected at TF time point. Moreover, the potential role of unknown mutations was explored through co-variation analysis and next-generation sequencing (NGS).

## Materials and methods

### Study design and participants

Two thousand and thirty-four HIV-1 CRF01_AE-infected patients were selected from a long-term ART cohort (follow-up every 3 to 6 months) at the First Affiliated Hospital, China Medical University in Shenyang between January 2002 and December 2017. Partial HIV-1 *pol* sequences (HXB2: 2253–3269) obtained by Sanger sequencing based on HIV drug resistance genotyping assays [24] for each participant at baseline were used to analyze the natural polymorphisms of CRF01_AE. One thousand three hundred and thirty patients received first-line ART (two nucleoside reverse transcriptase inhibitors [NRTIs] + one NNRTI), of which 105 patients experienced TF, defined by a persistently detectable viral load exceeding 1000 copies/ml after 6 months of ART according to the Consolidated Guidelines on the Use of Antiretroviral Drugs for Treating and Preventing HIV Infection of WHO in 2016 [25]. Forty-two TF patients receiving tenofovir/lamivudine/efavirenz (TDF/3TC/EFV) treatment, the first-line ART regimen in China, were further selected to analyze the acquired DRM profile of CRF01_AE, based on the detection of at least one major DRM (Stanford HIVdb algorithm v8.8) in Sanger sequencing involving HIV drug resistance genotyping assays. The study was approved by the Ethics Committee of the First Affiliated Hospital of China Medical University and all patients signed informed consent forms. The flow chart of participant selection and analysis is shown in Additional file 1: Figure S1. Data on the demographic and clinical characteristics of all participants were collected from clinical records and are shown in Additional file 2

### Phylogenetic and genotypic resistance analyses

For phylogenetic analysis, the *pol* sequences of 2034 CRF01_AE-infected patients at baseline were aligned with reference sequences downloaded from the Los Alamos HIV database (https://www.hiv.lanl.gov/) using the ClustalW tool in Mega v7.0 software, and then were manually edited. The models package in Mega v7.0 was used to determine the best nucleotide substitution model for this dataset. The reference sequences included twelve CRF01_AE strains from Africa and Thailand sampled between 1990 to 2001 and the representative sequences from seven major CRF01_AE lineages in China previously reported [17]. FastTree v2.1.9 was used to estimate an approximately maximum-likelihood phylogenetic tree based on the GTR + G + I nucleotide substitution model. The reliability of the phylogenetic tree was determined with local support values based on the Shimodaira–Hasegawa (SH) test with 1000 replicates. The phylogenetic tree was displayed using FigTree v1.4.3. Node SH-like support value ≥0.9 indicated a lineage [26].

A maximum-likelihood tree was reconstructed with the *pol* sequences of 42 TF patients at both baseline and TF using Mega v7.0. Bootstrap resampling (1000 datasets) of multiple alignments was performed to test the statistical robustness of the trees with the GTR + G + I nucleotide substitution model. A bootstrap value > 70 was identified as a cluster [27].

DRMs were identified using the Stanford University HIV Drug Resistance Database (https://hivdb.stanford.edu/) and interpreted using the Stanford HIVdb algorithm (HIVdb v8.8, Sierra v2.3.0; https://hivdb.stanford.edu/hivdb/by-mutations/).

### Polymorphism analysis

The mutation rates of amino acids at sites 1 to 240 of reverse transcriptase (RT) region of the *pol* gene were compared between the 2034 treatment-naïve CRF01_AE sequences and subtype B sequences from treatment-naïve patients in the Stanford University HIV Drug Resistance Database, with an average of 46,118 isolates (one isolate per person) analyzed at each site (https://hivdb.stanford.edu/cgi-bin/RTMutSummary.cgi; accessed on 04/08/2019). The mutation rates were also compared between 1148 TS patients and 105 TF patients. The HIV-1 strain HXB2 was used as the reference standard. The sites with a different amino acid (compared to the corresponding site in HXB2), and with a prevalence > 1%, were defined as natural polymorphism sites.

### Co-variation analysis

The CorMut package [28] v1.25.0 based on the R Project for Statistical Analysis (R v3.5.2) was used to analyze co-variation. The HIV-1 strain HXB2 was used as the reference sequence of location. Positively selected mutations (PSMs) were determined using selection pressure (Ka/Ks ratio), with Ka/Ks > 1 and log odds ratio (LOD) > 2 [29]. Conditional selection pressure (conditional Ka/Ks, cKa/Ks) was used to measure the correlation between PSMs, with cKa/Ks > 1 and LOD > 2 indicating the presence of directional co-variation.

### Temporal analysis of Y181C/ L228R mutations by deep sequencing

Longitudinal plasma samples between baseline and TF from four cases with Y181C and L228R mutations were selected. Viral RNA was extracted from the plasma samples using a QIAamp Viral RNA Mini Kit (Qiagen, Hilden, Germany) according to the manufacturer’s protocol and reverse transcribed using a Transcriptor First Strand cDNA Synthesis Kit (Roche Diagnostics, Indianapolis, IN, USA) with the specific primer Rev2–1 (5′-TCCTGCCATRGRAGATGCCTAA-3′). A 453-bp fragment (HXB2: 2868–3320) in the RT region of the *pol* gene was then amplified by two rounds of nested polymerase chain reaction (PCR) using a KOD-Plus-Neo kit (TOYOBO, Osaka, Japan) with the following outer and inner primers, respectively: MAW26/RT-21n (5′-GTATTTCTGCATTAAGTCTTTTGATGG-3′), 3-3F (5′-ACAGTACTAGATGTGGGAGATGC-3′)/3-3R (5′-TATATCATTGACAGTCCAGCT GTC-3′). The reaction conditions are shown in Additional file 3**.**

The PCR products were purified with Agencourt AMPure XP beads (Beckman Coulter, Brea, CA, USA) and then quantified using a Qubit 3.0 Fluorometer (Life Technologies, Carlsbad, CA, USA). The fragment length was accurately evaluated using an Agilent 2100 Bioanalyzer (Agilent Technologies, Waldbronn, Germany). Subsequently, the purified PCR products were adjusted to 2.5 ng/μl and indexed with an adaptor using a TruSeq Nano DNA LT library preparation kit (Illumina, San Diego, CA, USA) according to the manufacturer’s protocol. The indexed DNA libraries were analyzed using the Agilent 2100 Bioanalyzer and accurately quantified using a Roche LightCycler® 480 (LC480) Real-Time PCR system (Roche, Risch, Switzerland) and normalized to 10 nM, then pooled, denatured, and diluted to 15 pM, and finally mixed with 50% PHIX Control Libraries (Illumina, San Diego, CA, USA) to create a final volume of 600 μl.

Deep sequencing was performed using an Illumina MiSeq System (Illumina, San Diego, CA, USA). Oracle VM Virtual Box-5.2.22 software was used to build a virtual environment for running QIIME 2 Core-2018.4 (http://qiime.org/) in Windows operating system. According to the data quality assessment using FASTQC v0.11.7 software, paired-ended sequences were trimmed by 10–15 bp and truncated to 280–285 bp, and the other parameters were set to the default values. The data were denoised and dereplicated using dada2 plugin v2018.4.0 [30]. The sequences and numbers of HIV-1 quasispecies in each sample were reported with feature-table plugin v2018.4.0, and were then aligned using the ClustalW tool in Mega v7.0.

### Statistical analysis

The mutation rate of each amino acid site in RT was compared between treatment-naïve CRF01_AE and subtype B, between TS and TF CRF01_AE-infected patients, and between baseline and TF time point in 42 TF patients using the binomial distribution. The mutation rates and the number of DRMs of the 42 CRF01_AE TF patients between baseline and TF were also compared using the McNemar test and the Wilcoxon test, respectively. The statistical calculations were performed using SPSS software v20.0. *P* < 0.05 was used as the cutoff for significance**.**

## Results

### Transmitted drug resistance mutations and natural polymorphisms of CRF01_AE before treatment

**Fig. 1:**
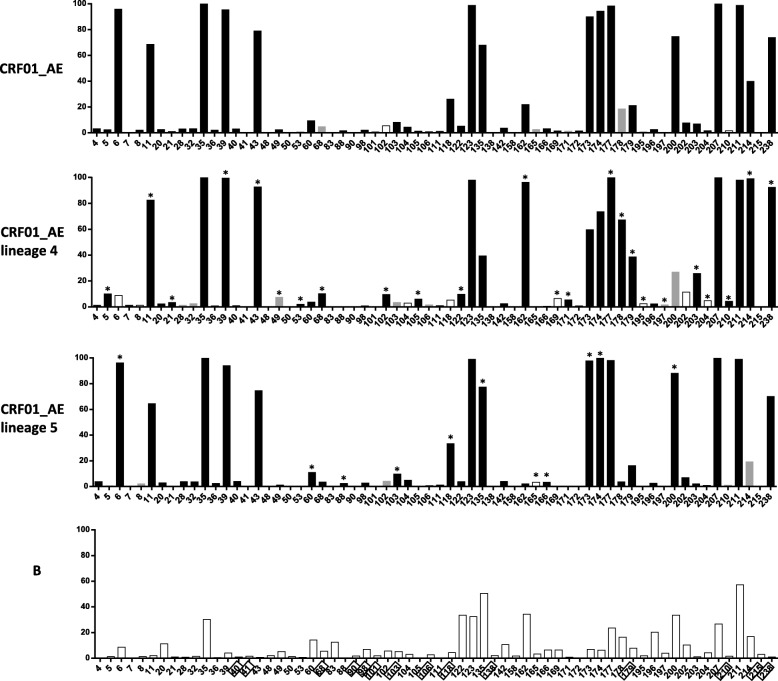
Natural polymorphisms in HIV-1 CRF01_AE. Positions are shown along the x-axis, and the mutation frequency for each subtype or lineage is shown along the y-axis. Sites associated with drug resistance in subtype B are boxed. Bar colors denote statistical significance: black is statistically significant (|Z value| ≥ 3); gray is borderline statistically significant (1 ≤ |Z value| < 3); white is not statistically significant (|Z value| < 1). The difference between CRF01_AE lineages 4 and 5 is marked with *, marked on the higher bars in lineage 4 or 5, respectively

In this study, 40 out of 2034 (1.97%) treatment-naïve CRF01_AE-infected patients had transmitted DRMs, with the common DRMs comprising K103 N, G190S, K101E, T215S, K65R, and K219Q. In addition to above DRMs, natural polymorphisms of amino acids with a prevalence > 1% were detected at 53 (53/240, 22.1%) sites in RT, of which nine sites (40, 68, 69, 98, 103, 118, 179, 210, and 238) were known drug resistance-associated sites. Moreover, 31 sites (4, 5, 6, 8, 11, 28, 32, 35, 36, 39, 40, 43, 88, 103, 104, 105, 111, 118, 123, 135, 172, 173, 174, 177, 179, 200, 203, 207, 211, 214, and 238) in CRF01_AE had higher mutation rates than subtype B HIV-1 strains in the Stanford HIV Drug Resistance Database (|Z value| ≥ 3) (Fig. 1). These 31 sites were defined as CRF01_AE-specific polymorphism sites, which included five known drug resistance-associated sites, site 238 (73.8%), site 118 (26.1%), site 179 (21.2%), site 103 (8.1%) and site 40 (3.1%), as well as 26 other sites that were not known to be associated with drug resistance (Fig. 1).

According to the phylogenetic analysis, the 2034 sequences mainly belonged to two CRF01_AE lineages, including 416 (20.5%) sequences of lineage 4 and 1522 (74.8%) sequences of lineage 5 (Additional file 4**:** Figure S2). Fifty-one and forty-four natural polymorphism sites in lineages 4 and 5 were detected, respectively, with differences in 35 sites between the two lineages (|Z value| ≥ 3). Both lineages had 26 polymorphism sites with higher mutation rates than in subtype B HIV-1globally (|Z value| ≥ 3), including two known drug resistance-associated sites (sites 179 and 238) (Fig. 1).

### Natural polymorphisms of CRF01_AE had little impact on treatment outcomes

**Fig. 2:**
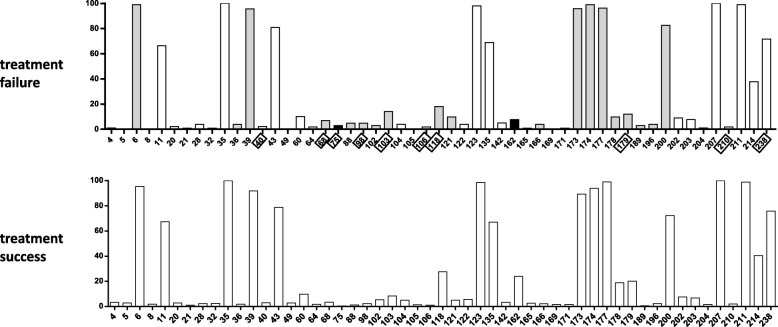
Natural polymorphisms compared between CRF01_AE-infected patients who experienced antiretroviral treatment (ART) failure and success. Positions are shown along the x-axis, and the mutation frequency for each group is shown along the y-axis. Sites associated with drug resistance in subtype B are boxed. Bar colors denote statistical significance: black is statistically significant (|Z value| ≥ 3); gray is borderline statistically significant (1 ≤ |Z value| < 3); white is not statistically significant (|Z value| < 1)

A total of 1330 out of 2034 CRF01_AE-infected patients received first-line ART, among which 105 (7.9%) patients experienced TF. We found 13 sites with differences between TF and TS patients (1148, 86.3%), comprising the polymorphisms at sites 75 and 189, which were only found in TF patients, and the polymorphisms at sites 4, 5, 8, 21, 32, 49, 105, 165, 169, 171, and 204, which were only found in the TS patients. The mutation rate of site 75 in TF patients was significantly higher than in TS patients (|Z value| ≥ 3) (Fig. 2).

### Common DRMs and potential new DRMs developed in CRF01_AE-infected patients with TDF/3TC/EFV TF

Forty-two CRF01_AE-infected patients with TDF/3TC/EFV TF were selected according to the flow chart presented in Additional file 1: Figure S1 to determine the acquired DRM profile of CRF01_AE. The time between baseline and TF sampling time point among the 42 TF patients was 184 days (interquartile range: 177.0–236.5). The number of DRMs at TF time point were significantly increased compared to baseline (Z = -5.604, *p* < 0.001). The sequences of the baseline and TF time point from each patient of the 42 TF patients clustered with bootstrap value higher than 85 in the phylogenetic tree (Additional file 5: Figure S3). The mutation rates of 14 sites increased significantly at TF time point, with increase ranging from 9.5 to 66.7% (Table 1). Of these 14 sites, 13 were known drug resistance-associated sites, including seven NRTI-associated sites and six NNRTI-associated sites. The NRTI-associated DRMs detected at TF time point in descending order included K65R (57.1%), M184 V/I (47.6%), S68G (26.2%), A62V (14.3%), K70E/R (9.5%), and Y115F (9.5%). The NNRTI-associated DRMs detected at TF time point included G190S/C (66.7%), K101E/N/Q (52.4%), V179D/I/A/T/E (45.2%), Y181C (42.9%), K103R/N/S (42.9%), and V106 M (23.8%) (Table 1). It was noted that an unknown mutation (V75 L) was detected at site 75, a drug resistance-associated site, which increased from 4.8% at baseline to 16.7% at TF time point (Z value = 2.494, *p* < 0.05; p _McNemar test_ = 0.008). Moreover, a new mutation (L228R) was detected at site 228, a non-DRM site in the Stanford HIVdb algorithm, which increased from 0% at baseline to 11.9% at TF time point (Z value = 2.306, *p* < 0.05; p _McNemar test_ = 0.063). We speculated that both V75 L and L228R might be potential new DRMs in CRF01_AE.

**Table 1:** Increase of mutation types and rates associated with failure of first-line treatment for HIV-1 CRF01_AE

Site of RT	B-WT		Mutation rate (%)		Increase (%)	Binomial distribution		McNemar test
		Mutations	Baseline	TF		Z value	p	p
62	A	V	0	14.3	14.3	2.542	**< 0.05**	**0.031**
65	K	R	0	57.1	57.1	5.797	**< 0.05**	**< 0.001**
68	S	G	4.8	26.2	21.4	2.715	**< 0.05**	**0.004**
70	K	E, R	0	9.5	9.5	2.049	**< 0.05**	0.125
75	V	L, I, A	4.8	23.8	19.0	2.494	**< 0.05**	**0.008**
101	K	E, Q, N	0	52.4	52.4	5.460	**< 0.05**	**< 0.001**
103	K	R, N, S	21.4	42.9	21.5	2.103	**< 0.05**	**0.004**
106	V	M	2.4	23.8	21.4	2.911	**< 0.05**	**0.012**
115	Y	F	0	9.5	9.5	2.049	**< 0.05**	0.125
179	V	D, I, A, T, E	26.2	45.2	19.0	1.822	> 0.05	**0.008**
181	Y	C	0	42.9	42.9	4.786	**< 0.05**	**< 0.001**
184	M	V, I	0	47.6	47.6	5.123	**< 0.05**	**< 0.001**
190	G	S, C	0	66.7	66.7	6.481	**< 0.05**	**< 0.001**
228	L	R	0	11.9	11.9	2.306	**< 0.05**	0.063

*B-WT* subtype B wild type, *TF* treatment failure. Boldface *P* values indicate *P* < 0.05. The boldface sites are known drug resistance-associated sites. The mutations listed are the types of mutations that occur at TF time point

### Relationships of potential new DRMs with known DRMs

To explore the role of potential new DRMs, the mutations at 14 sites with significantly increased mutation rates at TF were used for co-variation analyses. Nine known DRMs (K65R, V106 M, Y115F, V179 T/E/D, Y181C, M184 V, and G190S) and two potential new DRMs (V75 L and L228R) were demonstrated to be under positive selection pressure (Ka/Ks > 1, LOD > 2). Twenty-eight links were detected among these mutations (cKa/Ks > 1, LOD > 2) (Table 2). Among them, the known DRMs Y181C and G190S showed the strongest correlation (cKa/Ks_Y181C-G190S_ = 22.86, LOD = infinity). V75 L was correlated with known DRMs G190S (cKa/Ks_V75L-G190S_ = 3.24, LOD = infinity), K65R (cKa/Ks_K65R-V75L_ = 2.00, LOD = 5.04), and M184 V (cKa/Ks_V75L-M184V_ = 1.25, LOD = 4.03). L228R was correlated with known DRMs G190S (cKa/Ks_L228R-G190S_ = 2.25, LOD = infinity) and K65R (cKa/Ks_K65R-L228R_ = 2.00, LOD = 3.46), and strongly correlated with Y181C (cKa/Ks_Y181C-L228R_ = 6.00, LOD = 4.09) (Table 2).

**Table 2:** Co-variations of conditional selection pressure (cKa/Ks)

Mut1	Mut2	cKa/Ks	LOD	Mut1	Mut2	cKa/Ks	LOD
Y181C	G190S	22.86	Inf	K65R	Y115F	2	3.93
G190S	K65R	18	10.69	K65R	V179 T	2	Inf
G190S	Y181C	17	10.06	K65R	V179E	2	Inf
Y181C	**L228R**	6	4.09	K65R	**L228R**	2	3.46
V179D	V106 M	5	Inf	M184 V	Y115F	2	3.93
K65R	G190S	3.97	Inf	M184 V	V179D	2	9.16
K65R	Y181C	3.5	7.73	Y181C	V179 T	2	Inf
V75 L	G190S	3.24	Inf	G190S	Y115F	1.5	2.62
M184 V	V106 M	3	Inf	G190S	V179D	1.5	2.62
G190S	**V75 L**	2.5	4.03	Y181C	K65R	1.5	8.8
L228R	G190S	2.25	Inf	G190S	**L228R**	1.33	3.74
G190S	V179 T	2	Inf	M184 V	**V75 L**	1.25	4.03
G190S	V179E	2	Inf	K65R	V106 M	1.2	Inf
K65R	**V75 L**	2	5.04	Y115F	G190S	1.05	Inf

Boldface mutations have no annotation in the Stanford HIVdb algorithm. Inf: Infinity. Mut 1/2: mutation 1/2. Because the co-variation based on conditional selection pressure is directional, mutation 1 is the dominant mutation and mutation 2 is the affected mutation

### L228R occurred simultaneously or followed the appearance of Y181C

**Fig. 3:**
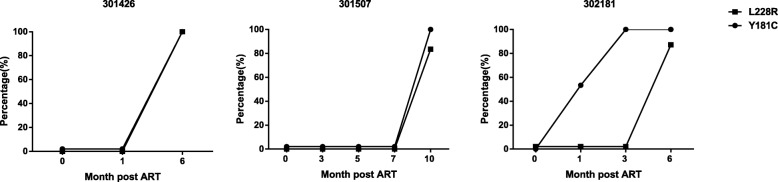
Temporal association of Y181C and L228R in CRF01_AE-infected individuals during antiretroviral treatment (ART). 301,426, 301,507, and 302,181 were three CRF01_AE-infected individuals in which both Y181C and L228R mutations were detected at treatment failure (TF) time point. Longitudinal plasma samples were studied using deep sequencing of the *pol*-RT sequences. Black circle represents the percentage of Y181C quasispecies; black square represents the percentage of L228R quasispecies

To further explore the temporal association and the evolutionary dynamics between Y181C and L228R, longitudinal plasma samples of four CRF01_AE-infected patients with Y181C and L228R mutations were studied using deep sequencing. The first case demonstrated a time lag between the Y181C and L228R mutations; Y181C occurred in 53.4% of the sequences at 1-month post treatment, which increased to 100% at 3 months post treatment, and L228R did not appear until 6 months post treatment, when 87.1% of sequences carried both Y181C and L228R mutations. The second and third cases had Y181C and L228R only at TF. For the second case, 100% of sequences carried both Y181C and L228R simultaneously while, for the third case, 80% of sequences carried both Y181C and L228R simultaneously, and the remaining 20% carried only Y181C (Fig. 3). The fourth case could not be analyzed due to sequencing failure.

## Discussion

To our knowledge, this study provides the most comprehensive information on HIV-1 drug resistance-associated natural polymorphisms and the acquired DRM profile of CRF01_AE in China to date using a large dataset from a long-term ART cohort in Shenyang. CRF01_AE isolates in this study demonstrated high levels of polymorphisms at both DRM sites and other sites, with several lineage-specific characteristics. However, the little variation in polymorphisms between TF and TS patients implied little impact of CRF01_AE polymorphisms on the development of DRMs. Moreover, this study found that the most common NRTI- and NNRTI-associated DRMs among CRF01_AE patients who experienced TF were similar to the DRMs among subtype B patients. However, several potential new DRMs selected during ART might be CRF01 _AE-specific DRMs. Among these DRMs, L228R occurred simultaneously or following the appearance of Y181C, and it might be an accessory mutation to Y181C.

In this study, 31 CRF01_AE-specific polymorphism sites (including five known DRMs) were detected, which is even higher than the number of polymorphism sites in CRF01_AE strains mainly from Southeast Asia in a global study on non-B HIV-1 over 10 years ago [31]. Moreover, the two CRF01_AE lineages in this study corresponded to the two lineages epidemic mainly among men who have sex with men (MSM) in China [17, 32], and they demonstrated both common and lineage-specific polymorphisms. A similar situation also occurred for *gag* and *env* regions of different lineages of CRF01_AE [33], suggesting that other lineages of CRF01_AE in China might have distinct polymorphisms, which might further complicate DRM development and drug resistance genotype interpretation [34].

In this study, the polymorphisms at five known drug resistance-associated sites (V179I/D, V118I, K103R, K238R, and E40Q) were polymorphic accessory mutations or other mutations that did not independently decrease drug sensitivity. None of them were associated with TF. Only V75 L, a low-frequency mutation, was associated with virologic failure, implying that most polymorphisms in CRF01_AE seldom lead to TF. A study on a London cohort in the United Kingdom found that different baseline polymorphisms, including V90I, A98S, and K103R, were associated with virologic failure [35], but their effects could not be differentiated from the impacts of the different treatment regimens and HIV strains.

At present, two NRTIs plus an integrase strand transfer inhibitor (INSTI) are recommended as a first-line ART regimen for adults in developed countries while, in developing countries like China, two NRTIs plus an NNRTI are still recommended as a first-line ART regimen. In this study, we evaluated the DRM profile of CRF01_AE after TDF/3TC/EFV TF. The most common acquired DRMs among CRF01_AE were K65R, M184 V, G190S/C, Y181C, and K103R, all of which are also common among subtype B and other subtypes. Despite some differences in the mutation type and rate, we cannot confirm that the differences were caused by the various subtypes [36, 37].

More importantly, we detected two mutations with significant increases but without annotation in the Stanford HIVdb algorithm, V75 L and L228R. Site 75 is a drug resistance-associated site but no explanation for V75 L is provided in the Stanford HIVdb algorithm. The V75 L mutation has been reported to provide a selective advantage by allowing escape from the host immune responses [38] and it is believed to be a TDF-associated mutation [23]. The L228R mutation has been reported to be related to the treatment of non-B HIV-1 subtypes in several studies [39, 40], but its phenotype has not yet been described. In this study, for the first time, it was suggested that L228R might be associated with the known DRM Y181C and it might act as an accessory mutation to Y181C based on a co-variation analysis and longitudinal evolution study. These results implied that more unannotated mutations in non-B HIV-1 during TF might be accessory mutations associated with drug resistance. Therefore, more studies are needed to strengthen the phenotypic research on drug resistance in non-B HIV-1 [41], and to provide more evidence for drug resistance interpretation for non-B HIV-1.

This study had several limitations. First, due to the high success rate of ART in this cohort, only a small number of TF patients could be included in the acquired DRM analysis. Second, the impact of the L228R mutation with or without Y181C needs further validation using virus growth competition and drug resistance phenotype assays. Nevertheless, this study provided more evidence of polymorphisms and DRMs in the non-B HIV-1 strain CRF01_AE.

## Conclusions

In summary, the high levels of polymorphisms in CRF01_AE had little impact on treatment outcomes, but some unknown mutations associated with TF might be minor DRMs. The results of this study indicate the need for more studies on drug resistance in non-B HIV-1, especially phenotypic studies to strengthen the drug resistance genotype interpretation, and to improve ART efficacy and minimize the transmission of drug-resistant strains.

## Supplementary information

**Additional file 1:** **Figure S1.** Flow chart of selection and analysis.

**Additional file 2:** Demographic and clinical characteristics of participants in this study.

**Additional file3:** Reaction conditions of cDNA synthesis and amplification of the target fragment.

**Additional file 4: Figure S2.** Phylogenetic analysis of the CRF01_AE HIV-1 *pol* sequences.

**Additional file 5:** **Figure S3.** Phylogenetic analysis of the 42 TF CRF01_AE-infected patients.

## Data Availability

The datasets used and/or analyzed during the current study are available from the corresponding author on reasonable request.

## Abbreviations

ART: Antiretroviral therapy
CRF: Circulating recombinant form
DRMs: Drug resistance mutations
ETR: Etravirine
INSTI: Integrase strand transfer inhibitor
LMICs: Low- and middle-income countries
LOD: Log odds ratio
MSM: Men who have sex with men
NGS: Next-generation sequencing
NNRTIs: Non-nucleoside reverse transcriptase inhibitors
NRTIs: Nucleoside reverse transcriptase inhibitors
PCR: Polymerase chain reaction
PSMs: Positively selected mutations
RPV: Rilpivirine
RT: Reverse transcriptase
TDF/3TC/EFV: Tenofovir/lamivudine/efavirenz
TF: Treatment failure
TS: Treatment success
WHO: World Health Organization
