# Supplementary figures and images for: Natural polymorphisms in HIV-1 CRF01_AE strain and profile of acquired drug resistance mutations in a long-term combination treatment cohort in northeastern China
Source: BMC Infect Dis. 2020 Feb 26;20:178. doi: 10.1186/s12879-020-4808-3 (PMC7045473; doi:10.1186/s12879-020-4808-3)

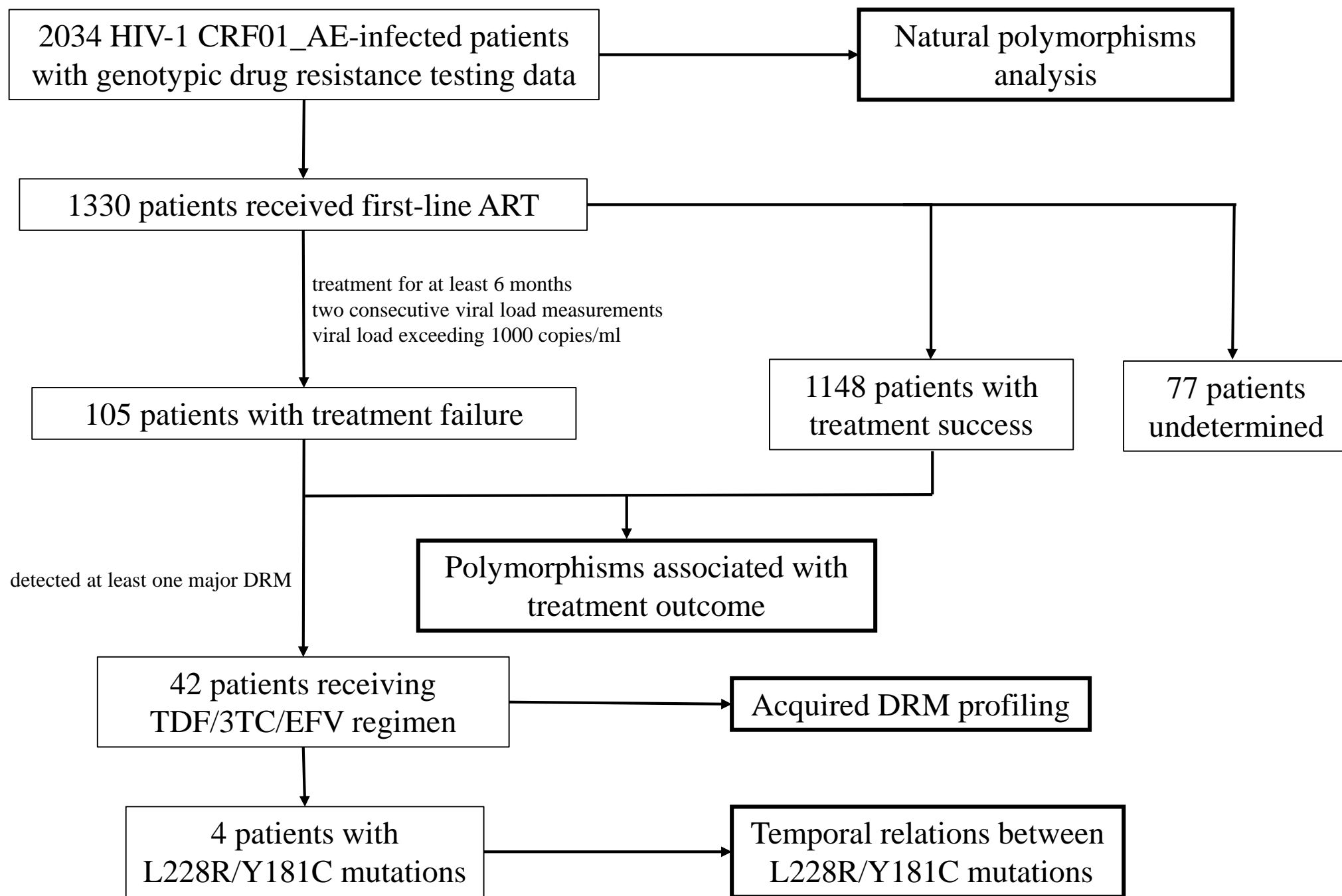

**Figure S1. Flow chart of selection and analysis**

Supplement: Supplementary file 1 — Additional file 1: Figure S1. Flow chart of selection and analysis. [file 12879_2020_4808_MOESM1_ESM.pdf]
