# Supplementary material for: Natural polymorphisms in HIV-1 CRF01_AE strain and profile of acquired drug resistance mutations in a long-term combination treatment cohort in northeastern China
Source: BMC Infect Dis. 2020 Feb 26;20:178. doi: 10.1186/s12879-020-4808-3 (PMC7045473; doi:10.1186/s12879-020-4808-3)
Supplement: Supplementary file 2 — Additional file 2: Demographic and clinical characteristics of participants in this study. [file 12879_2020_4808_MOESM2_ESM.pdf]

Table S1. Demographic and clinical characteristics of the 2034 CRF01_AE infected cases

Characteristics	total	%	Characteristics	total	%
Gender			Marital status		
male	1920	94.4	single/divorced/widowhood	1448	71.2
female	101	5.0	married	566	27.8
NA	13	0.6	NA	20	1.0
Age(years)			Degree of education		
Mean±SD	40.7±13.1		bachelor degree and above	463	22.8
>50	487	23.9	junior college	403	19.8
30-50	1107	54.4	high school or technical secondary school	440	21.6
20-30	422	20.7	primary or junior high school	582	28.6
<20	7	0.3	illiterate	3	0.1
NA	11	0.5	NA	143	7.0
Route of transmission			Baseline viral load (log_{10}copies/ml)		
homosexual	1699	83.5	Mean±SD	5.2±5.8	
heterosexual	255	12.5	>5	593	29.2
IDU	21	1.0	4-5	1106	54.4
others	2	0.1	3-4	244	12.0
NA	57	2.8	<3	17	0.8
			NA	74	3.6
Ethnicity			Baseline CD4+ count (cells/μl)		
Han	1739	85.5	Mean±SD	260.0±171.3	
Man	199	9.8	>500	151	7.4
minorities	77	3.8	200-500	1083	53.2
NA	19	0.9	<200	735	36.1
			NA	65	3.2

Note: NA means not available. SD: Standard Deviation. IDU: Intravenous Drug Use.

Table S2. Demographic and clinical characteristics of the 42 CRF01_AE infected cases

Characteristics	total	%	Characteristics	total	%
Gender			Marital status		
male	40	95.2	single/divorced	24	57.1
female	0	0	married	13	31.0
NA	2	4.8	NA	5	11.9
Age(years)			Degree of education		
Mean±SD	38.1±9.5		bachelor degree and above	5	11.9
>50	8	19.0	junior college	12	28.6
30-50	25	59.5	high school or technical secondary school	7	16.7
<30	7	16.7	primary or junior high school	9	21.4
NA	2	4.8	NA	9	21.4
Route of transmission			Baseline viral load (log_{10}copies/ml)		
homosexual	27	64.3	Mean±SD	5.6±5.7	
heterosexual	6	14.3	>5	28	66.7
IDU	0	0	4-5	14	33.3
others	1	2.4	<4	0	0
NA	8	19.0			
			Baseline CD4+ count (cells/μl)		
Ethnicity			Mean±SD	74.1±101.3	
Han	33	78.6	>200	3	7.1
Man	3	7.1	100-200	7	16.7
minorities	1	2.4	50-100	8	19.0
NA	5	11.9	<50	24	57.1

Note: NA means not available. SD: Standard Deviation. IDU: Intravenous Drug Use.
