# Supplementary material for: Natural polymorphisms in HIV-1 CRF01_AE strain and profile of acquired drug resistance mutations in a long-term combination treatment cohort in northeastern China
Source: BMC Infect Dis. 2020 Feb 26;20:178. doi: 10.1186/s12879-020-4808-3 (PMC7045473; doi:10.1186/s12879-020-4808-3)
Supplement: Supplementary file 3 — Additional file3: Reaction conditions of cDNA synthesis and amplification of the target fragment. [file 12879_2020_4808_MOESM3_ESM.pdf]

The reaction conditions of cDNA synthesis and amplification of the target fragment

The one-step RT-PCR system and reaction conditions were as follows: PCR grade water 1ul, primer Rev2-1 (10nmol/ul) 2ul, RNA template 10ul, preheating at 65℃ for 10 minutes, then on ice at least 2 minutes. After that, added Transcriptor Reverse Transcriptase Reaction Buffer (5×) 4ul, protector RNase Inhibitor (40U/ul) 0.5ul, deoxynucleotide Mix (10mM each) 2ul, and Transcriptor Reverse Transcriptase (20U/ul) 0.5ul, incubated at 55℃ for 30 minutes, then 85℃ for 5 minutes, and the reaction was terminated on ice.

The first-round system of nest PCR was as follows: buffer for KOD (10×) 2.5ul, dNTP (2mM) 2.5ul, MgSO4 (25mM) 1.5ul, outer primers: MAW26/RT-21n (10um) each 0.75ul, cDNA template 2ul, enzymatic free water 14.5ul, KOD-Plus-Neo (1U/ul) 0.5ul. Cycling conditions were: a denaturation step of 2 min at 94 ◦C, 35 cycles of 10 s at 98℃, 30 s at 58℃, 1 min 30 s at 68℃, then a final extension step of 10 min at 68℃, and finally holding at 4℃.

The second-round system of nest PCR was as follows: 3 parallel holes per sample. Buffer for KOD (10×) 2.5ul, dNTP (2mM) 2.5ul, MgSO4 (25mM) 1.5ul, inner primer 3-3F/3-3R (10um) each 0.75ul, first-round reaction product 2ul, enzymatic free water 14.5ul, KOD-Plus-Neo (1U/ul) 0.5ul. Cycling conditions were: a denaturation step of 2 min at 94 ◦C, 30 cycles of 10 s at 98℃, 30 s at 55℃, 1 min 30 s at 68℃, then a final extension step of 10 min at 68℃, and finally holding at 4℃.

The target gene fragment of the second-round PCR amplification product was identified by 1% agarose gel electrophoresis.
