# Supplementary material for: Natural polymorphisms in HIV-1 CRF01_AE strain and profile of acquired drug resistance mutations in a long-term combination treatment cohort in northeastern China
Source: BMC Infect Dis. 2020 Feb 26;20:178. doi: 10.1186/s12879-020-4808-3 (PMC7045473; doi:10.1186/s12879-020-4808-3)
Supplement: Supplementary file 4 — Additional file 4: Figure S2. Phylogenetic analysis of the CRF01_AE HIV-1 pol sequences. [file 12879_2020_4808_MOESM4_ESM.pdf]

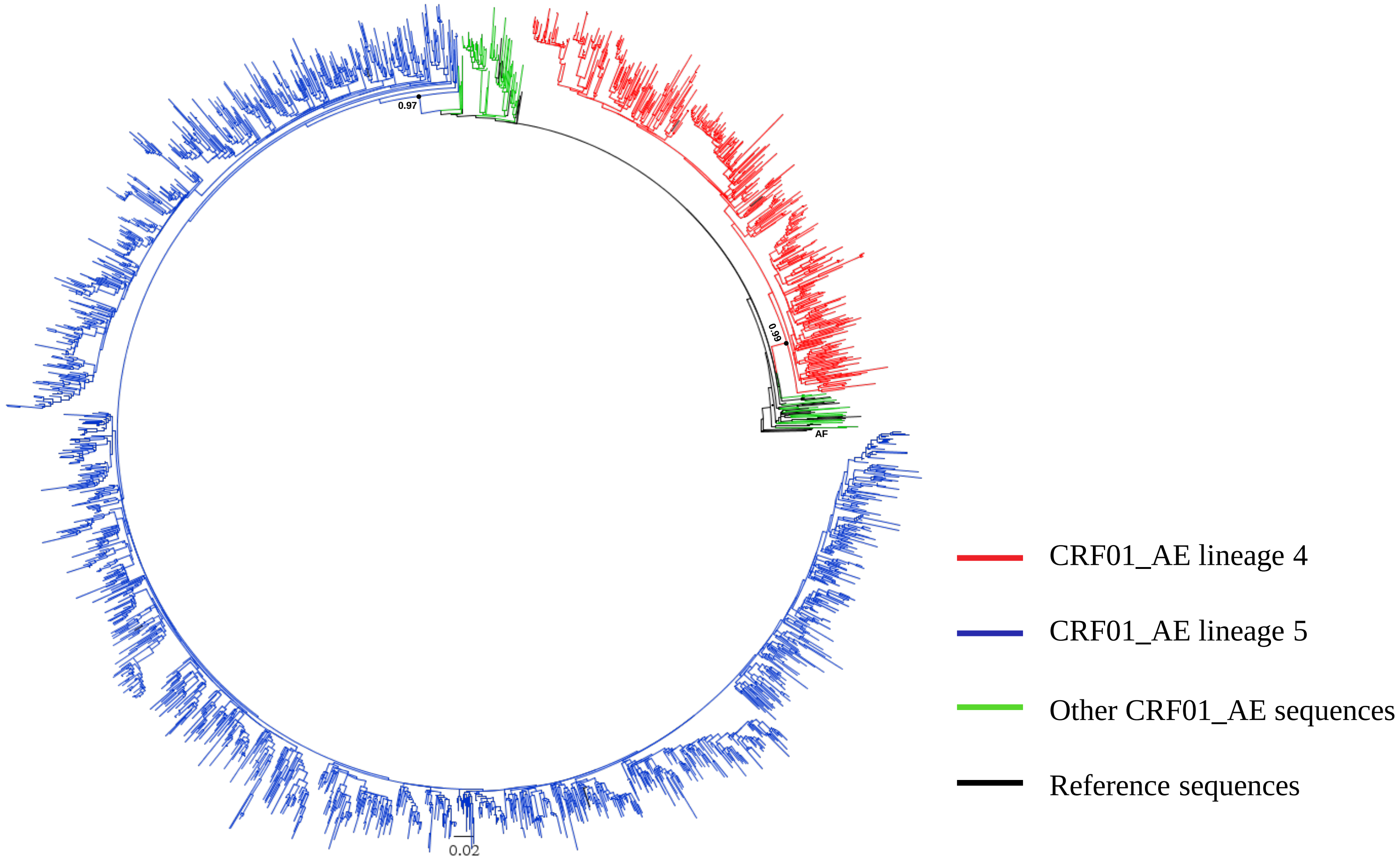

Figure S2. Phylogenetic analysis of the CRF01_AE HIV-1 pol sequences.

The *pol* sequences of 2034 CRF01_AE-infected patients at baseline were analyzed by the maximum-likelihood method in FastTree v2.1.9. The nucleotide substitution model was GTR+G+I. Local support values were computed by the Shimodaira-Hassegawa test (SH test) with 1000 replicates. Node SH-like support value ≥0.9 was identified as a lineage and was indicated at relevant nodes. Three African CRF01_AE reference sequences were at the root of the ML tree. The various lineages were color-coded.
