# Supplementary material for: Natural polymorphisms in HIV-1 CRF01_AE strain and profile of acquired drug resistance mutations in a long-term combination treatment cohort in northeastern China
Source: BMC Infect Dis. 2020 Feb 26;20:178. doi: 10.1186/s12879-020-4808-3 (PMC7045473; doi:10.1186/s12879-020-4808-3)
Supplement: Supplementary file 5 — Additional file 5: Figure S3. Phylogenetic analysis of the 42 TF CRF01_AE-infected patients. [file 12879_2020_4808_MOESM5_ESM.pdf]

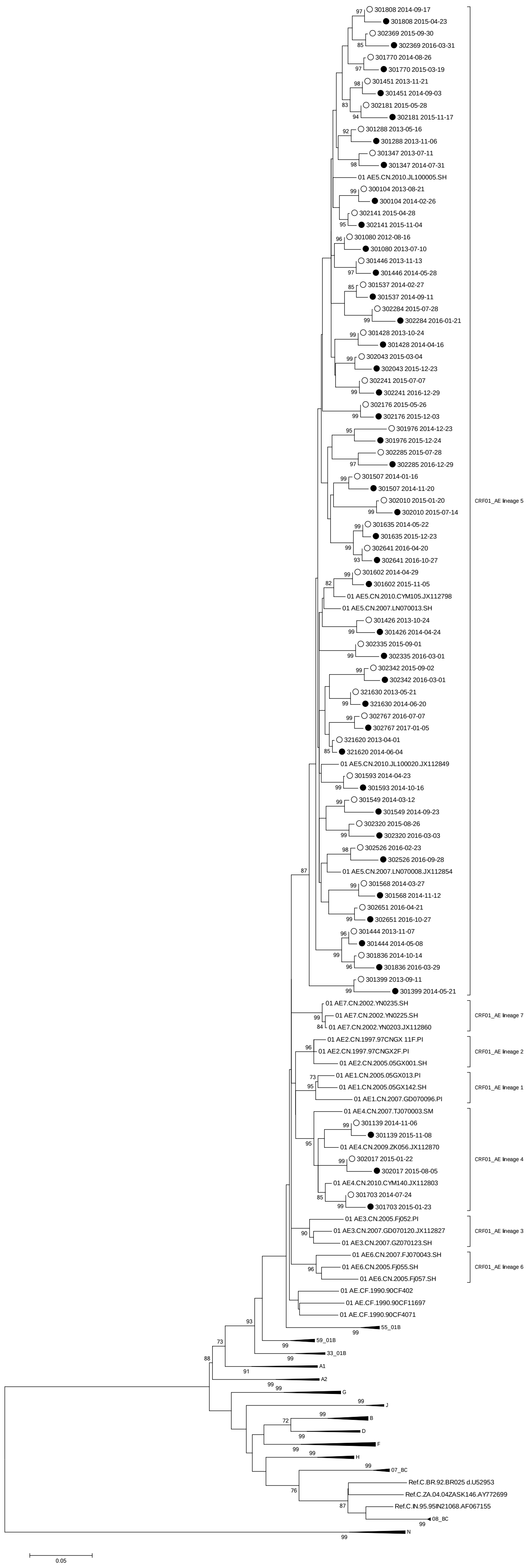

Figure S3. Phylogenetic analysis of the 42 TF CRF01_AE-infected patients.

The *pol* sequences of 42 CRF01_AE-infected patients at baseline and TF time point were analyzed by the Maximum likelihood method in Mega v7.0. The nucleotide substitution model was GTR+G+I. The bootstrap values of the 1000 replicates were labeled on the major branches. Bootstrap values <70 were hidden. The sequences at baseline were represented by hollow circles and the sequences at TF time point were represented by solid circles.
